# Annual Decline in Pentraxin 3 Is a Risk of Vascular Access Troubles in Hemodialysis Patients

**DOI:** 10.1155/2014/297954

**Published:** 2014-12-22

**Authors:** Kei Nagai, Atsushi Ueda, Chie Saito, Asako Zempo-Miyaki, Kunihiro Yamagata

**Affiliations:** ^1^Department of Nephrology, Faculty of Medicine, University of Tsukuba, 1-1-1 Tennodai, Tsukuba, Ibaraki 305-8575, Japan; ^2^Comprehensive Human Sciences, Faculty of Medicine, University of Tsukuba, 1-1-1 Tennodai, Tsukuba, Ibaraki 305-8575, Japan; ^3^Tsukuba University Hospital Hitachi Medical Education and Research Center, Jonan-cho 2-1-1, Hitachi, Ibaraki 317-0077, Japan

## Abstract

Pentraxin 3 (PTX3), a multifunctional modulator of the innate immunoinflammatory response, is higher in patients undergoing hemodialysis than healthy control. Our study focused on annual change in PTX3 levels in patients with chronic hemodialysis, because regularly undergoing hemodialysis for many years modifies vascular inflammatory status. To demonstrate whether annual change in PTX3 is associated with vascular events, we measured blood levels of pentraxins (PTX3 and high-sensitivity C-reactive protein (hsCRP)) at baseline and in the next year in 76 hemodialysis patients and observed 20 patients with vascular access troubles during follow-up years. The annual decline in PTX3, but not hsCRP, is a significant risk of the incidence of vascular access trouble that is a critical and specific complication for hemodialysis patients (hazard ratio; 0.732 per +1 ng/mL/year in PTX3, ^*^ *P* = 0.039). This study is the first to focus on the annual change of pentraxins in a hemodialysis cohort.

## 1. Introduction

Inflammation in patients with end-stage renal disease (ESRD) receiving hemodialysis (HD) is associated with malnutrition and cardiovascular diseases and resulted in poor clinical outcome [1]. Pentraxin 3 (PTX3) is a multifunctional soluble receptor that modulates the innate immunoinflammatory response and it belongs to the pentraxin-superfamily that includes C-reactive protein (CRP) [2, 3]. Plasma PTX3, similar to CRP, is considered to be an inflammatory marker of endothelial dysfunction and is also linked to increasing cardiovascular mortality risk [4]. However, because different cell types and organs produce PTX3 and CRP [2, 3], PTX3 and CRP may be involved in different pathophysiologic mechanisms [5]. The role of PTX3 has been demonstrated through experiments using PTX3-deficient or PTX3-overexpressing mice, and PTX3 may exert tissue-protective and anti-inflammatory effects [3, 6–9]. However, the functional role of human PTX3* in vivo* is still under discussion.

Boehme and colleagues first described that the PTX3 levels of HD patients with ESRD are higher than healthy subjects or ESRD patients without receiving HD [9]. It was interesting that spontaneous production of PTX3 in whole-blood samples from HD patients was significantly higher than that in samples from healthy subjects [9]. Otherwise, the mechanism underlying the production and the pathophysiological role of PTX3 in HD patients has not been elucidated fully. To address this issue, we focused on not only baseline PTX3 levels with a single measurement but also annual change in PTX3 levels in patients receiving chronic dialysis, because regularly receiving hemodialysis and continuously uremic condition for many years modify vascular status depending on several factors such as inflammation and oxidative stress [10, 11].

Here we show that the annual decline in PTX3, but not high-sensitive CRP (hsCRP), is a risk of the incidence of vascular access trouble that is a critical and specific complication for HD patients. This study is the first to focus on the annual change of pentraxins in a HD cohort.

## 2. Materials and Methods

### 2.1. Subjects and Sampling

This study was approved by the Ethics Committee of Namegata General Hospital, Namegata, Ibaraki, Japan, and nonhospitalized patients who regularly received HD were enrolled in this observational cohort study. All subjects were approached prospectively and gave informed consent to the study. Exclusion criteria were the presence of clinical signs of acute infection, active vasculitis, active hepatitis, and HIV at the time of evaluation and willingness to participate in the study. Of the 89 subjects initially enrolled in the study during 2011, 4 subjects died, and another 9 subjects did not complete exact one-year follow-up, primarily because they transferred to other hospitals, thus yielding a total of 76 patients who completed the study. Clinical demographic data of the subjects at baseline are given in Table 1. The patients received regular HD treatment 3 times/week and all of them have internal arteriovenous fistula (AVF) or arteriovenous graft (AVG) for hemodialysis. Blood was drawn from the arterial needle before starting a HD session both in baseline year (July 2011) and in the next year (July 2012) with exact one-year interval. Body mass index (BMI) was taken on a dialysis day immediately after a dialysis session. Vascular access troubles were defined as the need for catheter intervention or reoperation to remedy occlusion or stenosis of AVF or AVG or as the replacement of a permanent vascular catheter due to the occlusion of AVF or AVG from baseline year (July 2011) to two years later (the end of 2013). The patient, who died or transferred to other hospitals after measurement in next year, was censored at the time of death or lost to follow-up. Finally, the mean observed period in all subjects was 2.12 years.

### 2.2. Laboratory Measurements

Blood samples were placed in chilled tubes containing ethylenediaminetetraacetic acid (2 mg/mL) or no anticoagulants and centrifuged at 5500 g for 10 min at 4°C; the obtained plasma or serum, respectively, was stored at −80°C until analysis. Plasma concentrations of PTX3 were determined by using a commercial human PTX3 enzyme-linked immunosorbent assay (ELISA) kit system (Perseus Proteomics, Tokyo, Japan). Serum concentrations of hsCRP were determined by using the nephelometric N-latex CRP II kit (Siemens Diagnostics, Erlangen, Germany). Serum level of intact parathyroid hormone was determined by using Elecsys immunoassay systems (Roche Diagnostics, Basel, Switzerland). *β*2-microglobulin was determined by latex coagulation analysis kit (Eiken Chemical, Tokyo, Japan). Blood count was performed by autoanalyzer (Abbott Japan, Chiba, Japan). Other biochemical parameters were evaluated by using an autochemical analyzer (ci 16200, Toshiba, Japan).

### 2.3. Statistical Analyses

Values in tables are expressed as median with interquartile range (IQR). Statistical significance was set at *P* < 0.05. Comparisons between groups were appropriately assessed by the Mann-Whitney *U* test after normality test. Cox-regression models were performed to assess the risk of incidence of vascular access troubles; these models included age, sex, BMI, dialysis-periods, and presence of diabetes mellitus (DM). All analyses were performed by SPSS version 21.

## 3. Results

### 3.1. Comparison of Pentraxin Levels in Baseline Year among Subpopulations in This Study

Characteristic of this study population is shown in Table 1. In the 76 subjects, we observed 45 male patients, 31 patients with DM, 45 patients with age over 65 years, 42 patients with dialysis-period over 5 years, and 44 patients with BMI less than 22 kg/m^2^. We next examined differences in baseline levels of pentraxins (i.e., PTX3 and hsCRP) among subpopulations based on sex, presence of DM, age, dialysis-period, or BMI. It was shown that there were higher levels of PTX3 in patients with dialysis-period less than 5 years or BMI over 22 kg/m^2^ than the others (Table 2). However, levels of hsCRP are comparable among the subpopulations divided by sex, presence of DM, age, dialysis-period, or BMI (Table 2).

### 3.2. The Distribution of Annual Changes of PTX3 and hsCRP

**Figure 1:**
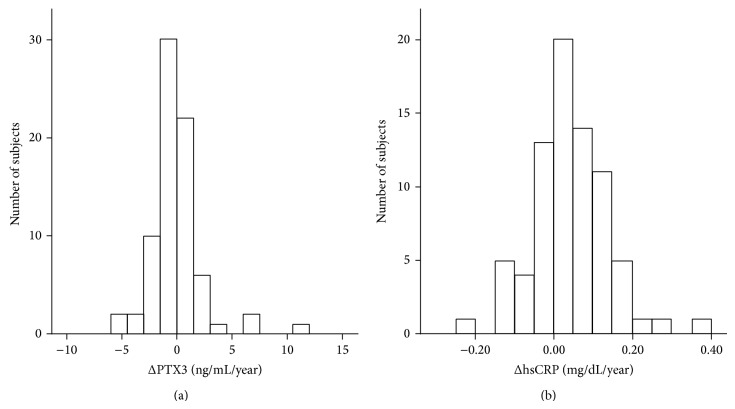
The annual change in pentraxins in the HD cohort. Distribution of annual change in PTX3 levels (ΔPTX3 (a)) or hsCRP (ΔhsCRP (b)) of the whole subjects is shown.

Because increased level of PTX3 would be dependent on length of dialysis-period as shown in Table 2, we observed the annual changes in levels of PTX3 and hsCRP from baseline to the next year in this study population (Figure 1). The mean change was +0.010 ng/mL/year or +0.032 mg/dL/year in PTX3 or hsCRP, respectively.

### 3.3. Annual Changes of PTX3 but Not hsCRP Are a Risk of Vascular Access Troubles

**Figure 2:**
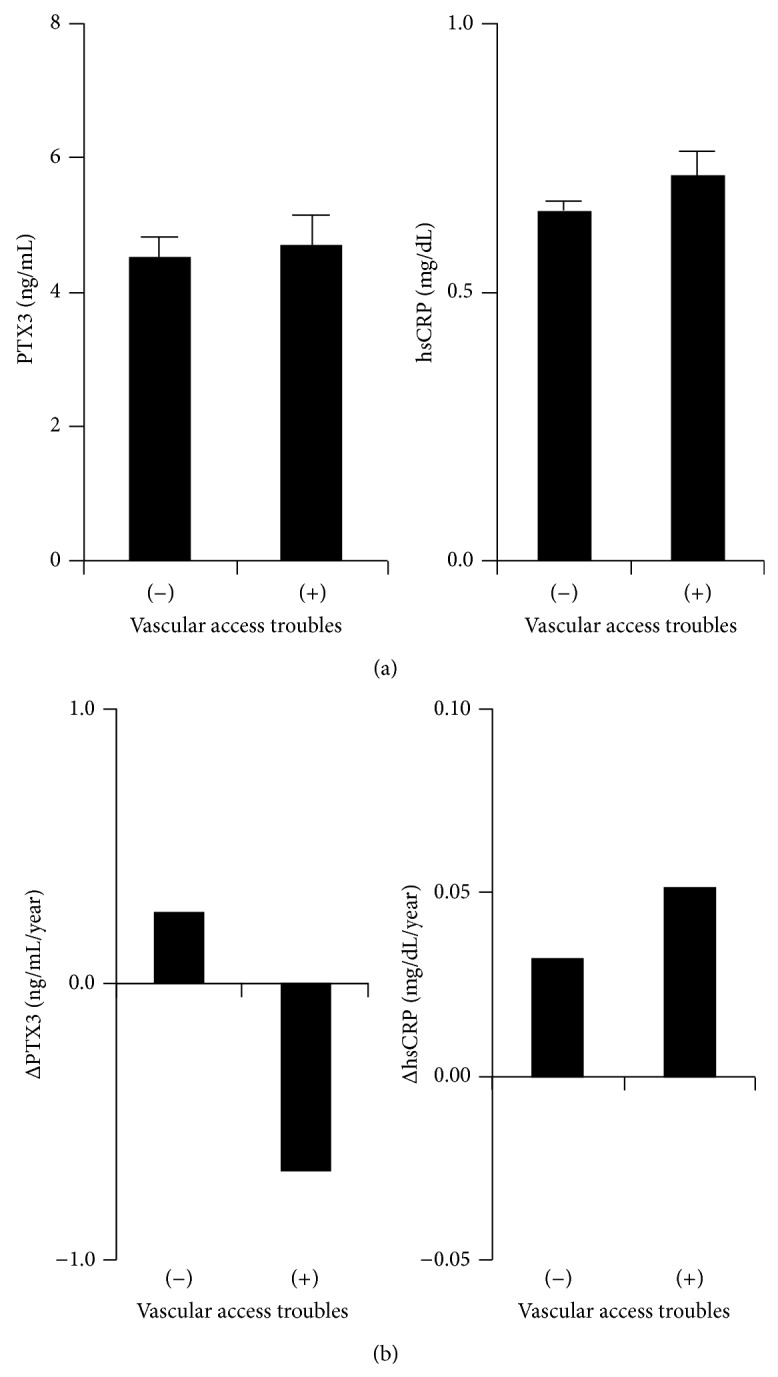
Baseline levels and annual change in pentraxins in subjects with vascular access troubles. Mean ± S.E.M. in baseline year (a) or annual change (b) of pentraxins in the subjects without or with vascular access troubles (− or +, *N* = 56 or 20, resp.) occurred during follow-up years. Significant difference of each parameter between the subjects without or with vascular access troubles was examined by Mann-Whitney *U* test.

To know the pathophysiological implications of annual change in pentraxin levels in HD patients, we examined whether pentraxins can predict the incidence of vascular events by utilizing cox-regression model. The number of patients with vascular access trouble during follow-up years (from 2011 to the end of 2013) was twenty. Table 1 shows the characteristics of the patients with stratification by the absence or presence of vascular access events. The level of PTX3 in baseline year in the patients with vascular access troubles is comparable to that without vascular access troubles as well as levels of hsCRP (Figure 2(a)). Interestingly, we observed the inverse trend for annual changes of parameters between hsCRP and PTX3 which is suggesting that annual decline in PTX3 would be related to the incidence of vascular access troubles (Figure 2(b)). Consistent with this result, Table 3 shows that annual decline in PTX3 is a significant risk for the incidence of vascular access troubles (hazard ratio; 0.732 per +1 ng/mL/year in PTX3, ^*^ *P* = 0.039) by cox-regression model with adjustment for covariants including dialysis-period and BMI which is crucially involving the level of PTX3, while hsCRP is not significant risk (hazard ratio; 3.605 per +1 mg/mL/year in hsCRP, *P* = 0.597).

## 4. Discussion

PTX3 is produced by various types of cells and increases rapidly in response to primary local inflammation and innate immunity [8, 9, 12, 13]. In patients with chronic kidney disease and those with ESRD receiving HD, increased, but not increasing, level of PTX3 is strongly predictive of poor clinical outcome and may be independent risk factors of mortality [4]. The subjects in our study had higher level of PTX3 at baseline (mean ± SD; 4.6 ± 2.2 ng/mL) than that of healthy control (0.76 ± 0.2 ng/mL) referred to in the previous observation [9]. Moreover, plasma level of PTX3, but not hsCRP, in patients with longer dialysis-period over five years is higher than that in those with shorter dialysis-period (Table 2). Therefore, we expected that annual change of PTX3 levels while undergoing chronic dialysis has distinguishing role from that of hsCRP.

Recently, notable results were described in PTX3-deficient murine experiments; the elevation of PTX3 during cardiovascular diseases has recently been postulated to be a compensatory response to protect the body from inflammation [3, 7, 8]. Human PTX3 prevents cells from becoming apoptotic by inhibiting the activation of factor H or by eliminating apoptotic cells quickly before they can secrete proinflammatory factors* in vitro* [14]. In contrast, serum levels of CRP are well recognized as reflection on the generation of proinflammatory cytokines* in vivo*, which cause malnutrition and cardiovascular diseases via several canonical pathways and result in poor prognosis [11]. Taken together, one explanation for high level of PTX3 in patients with long dialysis-period is that HD patients with higher PTX3 live longer because they avoid overproduction of inflammatory factors.

Although cross-sectional studies with a single measurement of pentraxins are available [4, 9], few previous cohort studies focus on the annual changes in PTX3 levels in patients receiving chronic dialysis. To address this complicated accumulation of knowledge about PTX3, a cross-sectional study with a single measurement of PTX3 might not be suitable to assess causality in the observed associations. Therefore, we conducted a longitudinal cohort study in HD patients to know whether annual changes of pentraxin levels could be a risk of the incidence for vascular event in HD patients. Consequently, we here show that annual decline in PTX3 is a risk of the incidence of vascular access trouble with adjustment for physical status represented by BMI that is well-known confounding factor for increased PTX3 [15–17] (Table 2). Though the involvement of PTX3 in the patency of AVF was previously mentioned only briefly [18], our study is the unique observation to support compensatory response of PTX3 to protect the body from vascular thrombotic event.

PTX3 is produced by various stimuli including lipopolysaccharide and cytokines such as tumor necrosis factor and interleukin-1*β* [9, 12]. Moreover, the promoter region of human PTX3 contains binding sites for the redox-sensitive transcription factors including nuclear factor *κ*B, which is critically involved in the regulation pathway of inflammatory mediators in innate immunity [13, 19]. Increased oxidative stress occurs in HD patients [10] and is dependent on many factors including aging, loss of residual renal function, uremic conditions, and receiving regular HD—all of which were represented in our study cohort. Though we could not determine what the major factor for annual change in PTX3 in this HD cohort was, it was speculated that excessive oxidative-stress would be one of the causes for annually changing PTX3.

Recently, some report suggested a crucial protective role of PTX3 in thrombotic diseases. In acute myocardial infraction, depletion of intracellular PTX3 in neutrophils correlates with increased plasma levels and with platelet-neutrophil aggregates* in vivo* [20]. These phenomena can be explained as follows: PTX3 is released from neutrophils via several stimuli and binds to P-selectin on activated endothelial cells [21] and activated circulating platelets [20] and dampens their proinflammatory and prothrombotic actions [22], thus contributing to its cardioprotective effects in human [20]. In contrast, several prospective studies show that hsCRP levels are positively associated with the incidence of myocardial infraction, stroke, and venous thrombosis [23, 24]. In thrombophilic condition such as essential thrombocythemia and polycythemia, high rate of thrombotic event is observed in the high CRP levels or low PTX3 levels [25]. Altogether, it is considered that PTX3 antagonizes the thrombotic function of CRP likely through a reduction of vascular inflammation.

In this research, by serial measurements of levels of PTX3, here we show that the annual decline in PTX3, but not in hsCRP, is a risk of the incidence vascular access troubles that is a critical complication for HD patients. This is the noteworthy study to focus on the annual change in pentraxins in a HD cohort and to support the evidence for the function of PTX3 to protect bodies from vascular thrombotic events.

## Figures and Tables

**Table 1:** Characteristics of the subjects with stratification by absence or presence of vascular access troubles.

	Total (*N* = 76)		Absence (*N* = 56)		Presence (*N* = 20)	
	Median	IQC	Median	IQC	Median	IQC
Gender (% man)	60	—	64	—	45	—
Diabetes (%)	41	—	38	—	50	—
Age (years)	67	58–77	66	58–77	67	58–77
Dialysis-period (years)	5.4	2.3–9.4	5.8	2.8–9.6	4	2.0–7.4
Body mass index (kg/m^2^)	21.9	19.6–24.0	21.2	19.4–24.0	22.5	20.5–24.0
RAS-inhibitor usage (%)	80	—	82	—	72	—
Statin usage (%)	14	—	11	—	22	—
Total protein (g/dL)	6.6	6.3–6.9	6.5	6.3–6.8	6.6	6.2–7.0
Albumin (g/dL)	3.3	3.0–3.4	3.3	3.0–3.5	3.3	3.0–3.4
Calcium (mg/dL)	8.9	8.5–9.8	8.8	8.4–9.8	9	8.6–9.9
Inorganic phosphorus (mg/dL)	5.2	4.6–6.1	5.2	4.6–5.8	5.3	4.6–6.3
Total cholesterol (mg/dL)	165	139–182	167	139–182	158	138–181
LDL-C (mg/dL)	95	77–112	95	73–113	93	77–107
Triglyceride (mg/dL)	93	72–145	93	74–136	97	72–174
Intact parathyroid hormone (pg/mL)	109	70–157	114	75–159	79	48–130
Hemoglobin (g/dL)	11	10.2–11.5	11	10.2–11.5	10.9	10.0–11.9
Ferritin (ng/mL)	65.6	40.8–114.9	66.2	41.1–116.2	62.9	40.6–114.9
Serum iron (mg/dL)	63.5	48.5–84.8	64	48.5–87.8	62	47.3–74.8
*β*2-microglobulin (mg/L)	29.1	25.5–32.2	28.9	24.7–31.9	29.9	26.8–34.3
PTX3 (ng/mL)	4.2	3.1–5.4	4.1	3.1–5.4	4.2	3.1–5.8
hsCRP (mg/dL)	0.66	0.56–0.73	0.64	0.54–0.72	0.68	0.62–0.75

Data at baseline year was presented as median and interquartile range (IQC). RAS: renin-angiotensin system, LDL-C: low-density lipoprotein cholesterol, PTX3: pentraxin 3, and hsCRP: high-sensitivity C-reactive protein.

**Table 2:** Comparison of pentraxin levels in baseline year among the subpopulations.

	Mean	SD	Mean	SD	*P* value
Sex	Male (*N* = 45)		Female (*N* = 31)		
Pentraxin 3	4.627	2.722	4.466	1.27	0.731
hsCRP	0.658	0.126	0.685	0.182	0.474

DM	Non-DM (*N* = 45)		DM (*N* = 31)		
Pentraxin 3	4.946	2.422	4.005	1.83	0.058
hsCRP	0.675	0.18	0.661	0.096	0.656

Age	<65 years (*N* = 31)		≥65 years (*N* = 45)		
Pentraxin 3	4.447	1.898	4.64	2.459	0.701
hsCRP	0.661	0.15	0.674	0.153	0.71

Dialysis-period	<5 years (*N* = 34)		≥5 years (*N* = 42)		
Pentraxin 3	3.961	1.852	5.048	2.416	0.030^*^
hsCRP	0.66	0.127	0.676	0.168	0.641

Body mass index	≥22 kg/m^2^ (*N* = 32)		<22 kg/m^2^ (*N* = 44)		
Pentraxin 3	5.175	2.397	3.719	1.689	0.003^**^
hsCRP	0.649	0.178	0.683	0.127	0.36

Data at baseline year was presented. Values of pentraxin 3 or hsCRP are expressed by ng/mL or mg/dL. DM: diabetes mellitus; hsCRP: high-sensitivity C-reactive protein. ^*^ *P* < 0.05, ^**^ *P* < 0.01.

**Table 3:** Cox-regression models for the incidence of vascular access troubles with or without multivariable adjustment.

	Unadjusted			Adjusted by DM, sex, age, dialysis-period, and BMI		
	Hazard ratio	95% C.I.	*P* value	Hazard ratio	95% C.I.	*P* value
Baseline PTX3	1.006	0.836–1.211	0.951	1.237	0.936–1.635	0.136
ΔPTX3	0.849	0.680–1.061	0.151	0.732	0.544–0.985	0.039^*^
Baseline hsCRP	3.644	0.469–28.30	0.216	2.713	0.389–18.92	0.314
ΔhsCRP	9.486	0.108–833.2	0.324	3.605	0.033–394.7	0.592

The incidence of vascular access troubles during follow-up years was set as the outcome. Covariants examined in Table 2 were used for adjustment. ^*^ *P* < 0.05. C.I.: confidential intervals, ΔPTX3: annual change in pentraxin 3 (ng/mL per year), and ΔhsCRP: annual change in high-sensitivity C-reactive protein (mg/dL per year).
